# Avascular necrosis of head of femur

**DOI:** 10.4103/0019-5413.45316

**Published:** 2009

**Authors:** Philippe Hernigou

**Affiliations:** *University Paris XII, Hospital Henri Mondor, 94000 Creteil, France*

Osteonecrosis of the femoral head (ONFH) is a disabling condition that affects mainly younger subjects in the midst of their working lives; to this day, it has remained a devastating disease. It affects both hips in more than 60% of individuals and is being diagnosed with increasing frequency. Its treatment stands at the borderline between Medicine and Surgery and requires a thorough understanding of the pathogenesis, natural history, and treatment options as a function of the different disease stages, which are best assessed with further investigations. The management of hip osteonecrosis remains one of the more challenging problems facing the orthopaedic surgeons. Without definitive treatment, 70% to 80% of clinically diagnosed cases will progress, and will undergo arthroplasty. Our primary goal is to preserve rather than replace the femoral head and articular cartilage. There is no completely satisfactory method to accomplish this, although a number of approaches have been described. Opinions often vary as to their indications and effectiveness.

“Conservative” management with protected weight bearing is not effective. This treatment will allow progression of their condition in one or two years. Usually, the patients are kept off weight-bearing on the affected side; however, this treatment principle has several limitations: the lesion is frequently bilateral; it does not abolish the muscle tone around the joint, even when the patient is lying down. The use of two elbow crutches (as a substitute for axillary crutches) does not provide the required complete weight relief; and even if crutches afford some protection, there is still the question of how long the patients should be kept off weight-bearing.

Before the onset of femoral head collapse, nonoperative treatment includes the use of various electromagnetic, acoustic stimulation or pharmacologic agents. These are still in a stage of evaluation and development. Leo Massari et al have analysed the effects of stimulation with pulsed electromagnetic fields (PEMFs) on the treatment of avascular necrosis of the femoral head. Both hypothesized that the effect of PEMF stimulation may be to protect the articular cartilage from the catabolic effect of inflammation and subchondral bone-marrow edema.

Pain is the first symptom in osteonecrosis. Before collapse, pain may be in relation with bone marrow edema frequently seen on MRI in early stages of osteonecrosis. Roland Meizer has evaluated the efficacy of the vasoactive drug iloprost in the treatment of pain in bone marrow edema, which is sometimes a pre-condition of hip osteonecrosis and concluded that the use of parenteral iloprost might be a viable method for pain in this situation.

The repair of necrotic cancellous bone in osteonecrosis involves two different processes, which appear to occur independent of each other: cell proliferation and invasion of the femoral head by reparative tissue. Bone resorption is associated with the repair process and is in relation with osteoclasts. These cells are derived either from mesenchymal precursors or from blood monocytes. What causes a change in the mechanical properties of the femoral head as an organ, to make it deform and collapse, is the resorption due to osteoclasts involved in the reparative process. It would, therefore, make sense to stop the activity of these osteoclasts. Eli peled has evaluated the influence of alendronate treatment on the rat femoral head shape after 6 weeks of daily treatment, when compared with controls. Alendronate treatment prevented the distortion and destruction of the femoral head. Osteoclast inhibition might prolong the bone creeping substitution, which might reduce disability due to femoral head collapse.

Very early in the course of the disease, core decompression remains the most logical treatment modality if one accepts that the condition is a compartment syndrome with increased pressure inside the femoral head. Core decompression has been in use for a considerable time; the results reported by different authors vary no doubt because of different patient populations and different stages treated with core decompression. However, while fundamental research and clinical studies have shown that dead bone may be revascularized by living bone, the reparative osteogenic potential is slight in ONFH: because the number of bone progenitor cells in the uninvolved part of the femoral head and in the trochanteric region is less than in healthy subjects. It would, therefore, make sense not only to core, but to introduce new osteoprogentor cells. This can be done by placing a vascularized graft into the coring tract. Sudhir Babhulkar analyzed and reported a series of patients of osteonecrosis of femoral head treated by core decompression and vascular pedicle grafting of part of iliac crest based on deep circumflex iliac vessels. The core decompression and vascular pedicle grafting reduced the intraosseous tension to achieve early revascularization of ischemic femoral head. The high percentage of marrow and osteogenic cells survive within a vascularized pedicle graft, which helps in early vascularization and we have been able to achieve good outcome.

The same result may be produced more readily by harvesting bone marrow from the anterior iliac crests, concentrating the marrow thus obtained, and re-injecting it into the necrotic zones. Philippe Hernigou reported a series of patients operated on for early stages of osteonecrosis of the hip with autologous bone marrow. The aim was to increase the number of osteoprogenitor cells in the proximal femur to stimulate bone remodeling and creeping substitution. Hips with stage I osteonecrosis of the femoral head at the time of surgery demonstrated total resolution of osteonecrosis based on preoperative and postoperative MRI studies.

So, many different approaches have been described for the treatment of osteonecrosis. The decision as to which treatment to select will depend upon a number of factors, including a thorough clinical evaluation of the patient as well as the use of an effective method of staging to determine both the type and extent of the pathology present in the hip. Ramesh Kumar Sen described the different techniques that the surgeon may use according to the stage of the osteonecrosis.

We are pleased that it has been possible to devote this issue of the journal to the treatment of osteonecrosis of the femoral head. We have been fortunate in recruiting authors who have addressed their topics concisely and effectively. Our goal is to provide our readers with an informed and up-to-date overview of a challenging and poorly understood problem in order to assist them in the management of the patient with osteonecrosis of the hip. Challenges still lie ahead. In the coming years, we hope to conduct other studies on the development of animal models and on new therapeutic agents. Osteogenic and angiogenic stem cells or growth factors should improve the surgical techniques in the next years. We conclude by thanking the authors for their contribution to this special symposium. We also would like to express our sincere appreciation to the editor of the Indian Journal of Orthopaedics, for his support for this symposium.

